# Essential Medicines in a High Income Country: Essential to Whom?

**DOI:** 10.1371/journal.pone.0143654

**Published:** 2015-12-09

**Authors:** Mai Duong, Rebekah J. Moles, Betty Chaar, Timothy F. Chen, World Hospital Pharmacy Research Consortium (WHoPReC)

**Affiliations:** Discipline of Pharmacy Practice, Faculty of Pharmacy, The University of Sydney, Sydney, New South Wales, Australia; Ottawa Hospital Research Institute, CANADA

## Abstract

**Objective:**

To explore the perspectives of a diverse group of stakeholders engaged in medicines decision making around what constitutes an “essential” medicine, and how the Essential Medicines List (EML) concept functions in a high income country context.

**Methods:**

In-depth qualitative semi-structured interviews were conducted with 32 Australian stakeholders, recognised as decision makers, leaders or advisors in the area of medicines reimbursement or supply chain management. Participants were recruited from government, pharmaceutical industry, pharmaceutical wholesale/distribution companies, medicines non-profit organisations, academic health disciplines, hospitals, and consumer groups. Perspectives on the definition and application of the EML concept in a high income country context were thematically analysed using grounded theory approach.

**Findings:**

Stakeholders found it challenging to describe the EML concept in the Australian context because many perceived it was generally used in resource scarce settings. Stakeholders were unable to distinguish whether nationally reimbursed medicines were essential medicines in Australia. Despite frequent generic drug shortages and high prices paid by consumers, many struggled to describe how the EML concept applied to Australia. Instead, broad inclusion of consumer needs, such as rare and high cost medicines, and consumer involvement in the decision making process, has led to expansive lists of nationally subsidised medicines. Therefore, improved communication and coordination is needed around shared interests between stakeholders regarding how medicines are prioritised and guaranteed in the supply chain.

**Conclusions:**

This study showed that decision-making in Australia around reimbursement of medicines has strayed from the fundamental utilitarian concept of essential medicines. Many stakeholders involved in medicine reimbursement decisions and management of the supply chain did not consider the EML concept in their approach. The wide range of views of what stakeholders considered were essential medicines, challenges whether the EML concept is out-dated or underutilised in high income countries.

## Introduction

The concept of “essential medicines” dates back to military tradition, in which therapeutic supplies (such as penicillin) were essential to be carried by soldiers, field medics, and camp infirmaries, into combat zones. This was also applied to the rationalising of therapeutic restrictions necessary during wartime economy [1]. Ensuring access to essential medicines has been considered a basic human right, in line with access to food, water, shelter and education [2]. The Essential Medicines List (EML) was introduced by the World Health Organization (WHO) in 1977, as a core list of 186 pharmaceuticals deemed necessary to manage the disease burden and basic health needs of a population (Box 1) [1,3]. Today, the WHO’s Model List of Essential Medicines (WHO EML) includes 409 active substances, is updated every two years, includes low and high cost medicines, and is applied to all income settings in 156 countries [4–6].

**Box 1. The WHO Definition of Essential Medicines [3]**

“Essential medicines are those that satisfy the priority health care needs of the population. They are selected with due regard to disease prevalence, evidence on efficacy and safety, and comparative cost effectiveness. Essential medicines are intended to be available within the context of a functioning health systems at all times, in adequate amounts, in the appropriate dosage forms, with assured quality, and at a price the individual and the community can afford.”

Nearly forty years since the introduction of the WHO EML, few studies have investigated the impact of EML policies on access to medicines [7,8]. Although the EML concept appears simple, it can be complex to implement and maintain. Therefore, the intention of having access to essential medicines within the context of a functioning health care system remains a work in progress for many countries [6]. Challenges in managing EMLs are most apparent in low-to-middle income countries (LMICs), compared to high-income countries (HICs) with sophisticated health care systems and national health insurance schemes [9,10]. Furthermore, the Access to Medicines Gap reported by the WHO states that one third of the world’s population still does not have access to medicines, which rises to up to half of the population in some LMICs [11,12].

A study by Cameron et al [9] showed that despite national EML policies, LMICs still experience low availability of generic medicines and high prices paid by consumers. Within LMIC settings these challenges are further complicated by scarce resources, limited availability and substandard and/or counterfeit products which pose safety risks to consumers [9,13]. Meanwhile, studies have also shown discrepancies between the WHO EML and national medicines lists in middle to high income countries [14,15]. Whilst disparities exist between how countries apply the EML concept, the selection process and reimbursement of medicines remains highly contentious across both high and low income country settings [10,16–18].

In line with the EML concept, national reimbursement schemes aim to enable access to medicines for a population within a functioning health system [16]. They are often used around the world in publicly funded HIC health care systems with complex supply networks. These reimbursement recommendations and/or decisions made by medicine review committees in HICs apply health technology assessment (HTA) methods to assess the value of medicines, including consideration of therapeutic outcomes, cost effectiveness and availability of alternative treatments [16,19,20]. Examples of these committees are the Pharmaceutical Benefits Advisory Committee (PBAC) in Australia, Pharmac in New Zealand, the Scottish Intercollegiate Guidelines Network (SIGN) in Scotland, and the National Institute for Health and Care Excellence (NICE) in the United Kingdom.

Despite efforts to evaluate the usefulness of having EML policy [7,8], uncertainty around the definition of essential medicines and scrutiny of the WHO EML selection committee deliberations require further transparency [21–23]. Therefore, research is needed to understand issues influencing the EML decision making process and influences on stakeholders involved in this process, for both HIC and LMIC settings. The aim of this study was to explore the perspectives of a diverse group of stakeholders engaged in medicines decision-making around what constitutes an “essential” medicine, and how the EML concept functions in a high-income country context.

## Materials and Methods

Ethics approval was obtained from the Human Research Ethics Committee (HREC) at the University of Sydney. All study participants were provided the study information statement, consent form and brief interview guide prior to interviews. As required by HREC, written consent was obtained. Participants were guaranteed confidentiality and anonymity.

A qualitative semi-structured interview protocol was developed based on existing literature and research objectives (S1 Text) [24]. The study was reported in accordance to the COREQ-32 checklist criteria (S2 Text) [25]. The interview guide was pilot tested with a hospital pharmacist and researcher, and provided to participants prior and during interviews as part of the consent process. As part of the interview guide, participants were asked what their thoughts were on the EML, whether the concept of EML applied to the Australian context, and what makes a medicine essential. Australia was selected as a HIC setting, since it offers universal health coverage under the auspice of the National Health Act, making medicines available to the population at subsidised prices through the Pharmaceutical Benefits Scheme (PBS) [26]. Once a list of 139 free life-saving and disease-preventing medicines [27], the PBS now contains over 800 medicines, marketed in over 4,500 different brands [28,29]. This research is part of a broader international study on the management and supply of essential medicines.

Between October 2012 and January 2015, 32 Australian stakeholders were recruited for in-depth interviews through purposive and snowball sampling approaches [30,31]. Forty eight participants were contacted to participate in the study. Seven participants declined to participate either due to perceived limitation of expertise or time constraints. These participants however referred a colleague. A further nine failed to respond to the invitation to participate. Stakeholders represented in this study were working in government, regulatory bodies, hospital practice, the pharmaceutical industry, wholesale/distribution companies, medicines non-profit organisations, academia and consumer health groups. The study targeted individuals with EML experience, PBS reimbursement and selection knowledge, or those with experience managing drug shortages. Participants included were recognised as leaders, advisors and/or experts amongst colleagues or professional organisations in the area of quality use of medicines, medicines policy, medicine distribution, procurement management, manufacturing, or health economics. Most participants had professional backgrounds as physicians or pharmacists. However, consumer representatives, chief executive officers, supply chain managers, and health economists were also included. Some had broad experiences across multiple and overlapping sectors, and/or had international experience working with EMLs in LMICs.

In-depth face-to-face, teleconference and Skype interviews were conducted with participants. Interviews were conducted prior to the 19^th^ WHO EML, published in May 2015 [4]. Audio recordings of the interviews were de-identified, transcribed verbatim and secondary verification of the transcripts was conducted to ensure accuracy. De-identified supplementary field notes were included in the data analysis. Interviews were conducted until thematic saturation was achieved (when several participants repeated similar or recurrent concepts in their response), as described by Bazeley [32]. Interviews lasted a median time of 63 minutes (IQR: 50–71).

Transcripts and field notes were imported into the qualitative analysis software program N-Vivo 10 for coding and data management [33]. Sequential analysis was used to explore relevant issues according to participants’ responses [34]. A grounded theory approach was applied in data analysis to extract themes and key concepts using iterative constant comparative techniques [35]. The grounded theory approach is a well-established research method which has been widely accepted and supported as a high standard of analysis used in interpreting and reporting qualitative research [32,35–38]. Accordingly, open, axial and selective coding methods were used to identify and interpret topics, themes, and concepts [32].

One researcher independently conducted the open coding thematic content analysis to identify themes and concepts, followed by two researchers conducting axial coding together that was validated by the rest of the research team. Final selective coding was performed as a team. While the initial thematic content analysis was performed by one researcher, the continuous consultative approach offered reflexivity and explored relationships between these themes and concepts. Results derived from the grounded theory approach were applied to the comprehensive theory of collaboration to build a conceptual model [39]. As described by Patton [37], reflexivity of data collection, interpretation and analysis was offered by the researchers’ broad experiences working across multiple pharmacy, patient care and administrative settings, with international experiences in both HICs and LMICs. Participants were offered the opportunity to validate the accuracy and interpretation of their views expressed in selected quotes.

## Results

Participants had a broad range of views on the notion of what is meant by an “essential” medicine. Three main concepts were derived from the views of multiple stakeholders on what constituted an essential medicine and how the EML concept applied in the Australian context. Table 1 illustrates the corresponding quotes reported in the results.

**Table 1:** Stakeholder Comments on the Aim and Function of an EML in Australia.

Concept	Significance	Quotations
**1) The definition and function of an EML in Australia is interpreted differently amongst stakeholders.**	Access to essential medicines is a basic human right	*[Q1] “No one should live with infection*, *serious pain*, *{or} a disability that can be treated*. *The essential elements of healthcare should be available to everyone whether you live in rural Australia*, *the city*, *on a good wage or without a job*. *Everybody has a right to that basic level of healthcare*. *That includes access to drugs*.*” (Participant 31- Consumer)*
	The notion of an “essential” medicine has evolved	*[Q2] “When the EML was created*, *it was about the aspirin(s)*, *{and the} penicillins*.* *.* *. *things that really were going to make a difference*. *These days*, *it’s no longer the case*. *You have to consider is this good value for money*? *I think the essential medicines list has evolved and changed*. *(Participant 25- Academic)*
	National reimbursement of medicines through the Australian PBS is a functioning EML	*[Q3] “Ours {PBS list} is bigger than the WHO list*, *but that’s appropriate for a wealthy country like Australia… yet it certainly does only encompass a fraction of all the drugs available*. *It {the PBS} is a limited list*, *selected on the basis of diseases in the country and cost effectiveness*, *and that’s the principle of the WHO list*.*”(Participant 22-Government)*
	There lacks a clear distinction between reimbursed medicines and essential medicines.	*[Q4] “I would like to think {the PBS} is an EML*, *otherwise why are we funding them*? *One of the things we need to do better in Australia is to remove drugs which have been superseded by other drugs as far as their effectiveness or cost effectiveness is concerned*.*” (Participant 30-Consumer)*
		*[Q5] “You tend to get a wish list*, *and then it becomes difficult to sort through what really is essential and what’s would be nice to have*. *(Participant 23- Non-profit)*
	An EML can support stakeholders to sustain fundamental health services.	*[Q6] “Today*, *the EML is the PBS by default*. *It's far from essential… When securing supplies*, *we really need to identify what is essential*, *and why it's essential*. *If it's not essential then acknowledge that it's not*. *So that in my day-to-day practice*, *drugs that I rely on to keep patients alive are available*.*” (Participant 6*, *Healthcare Provider)*
	Focus on medicines cost and cost-effectiveness diverts from the notion of essential	*[Q7] “The WHO defines essential medicines in such a way that cost effectiveness is one of the criteria*. *I think that’s a perversion of the idea of an essential medicines list*. *A medicine is intrinsically essential*. *You either need it or you don’t*. *The cost of that medicine is not a dimension of your need or the potential benefit you may derive from that medicine*.” (*Participant 14*– *Government)*
**2) The selection criteria of medicines and the context to which the EML concept is applied are dynamic variables which influence decision making.**	A medicine is essential at the point of care for the individual	*[Q8] “{Essential medicines} are life-saving*, *{or} enable management of a difficult*, *chronic condition*. *The individual consumer of course wants access to the drugs which will help their individual conditions and needs*.*” (Participant 30- Consumer)*
	A reimbursement system assesses cost effectiveness and additional benefit of a medicine for some individuals not the essential need of a medicine for a population.	*[Q9] “The PBS is different*, *because they’re not essential by definition*. *They are {medicines} that have been proven to be cost effective and the government is willing to pay to give their citizens access to these medications*. *Some could be lifesaving*, *high-cost drugs*, *and then that’s a different program*. *The structure in a country like Australia is different because it’s how much you’re willing to pay for an extra innovation*. *So the concept is different from an EML*.*” (Participant 25-Academic)*
	Perception that an EML is only used in low middle income settings	*[Q10]* “*The danger is that you end up saying that an EML is what less well-off countries have and reimbursement lists are what wealthier countries have*. *“(Participant 20-Academic)*
	The supply of essential medicines is vulnerable to disruptions in all country settings.	*[Q11] “We live in a complex world where some pharmaceutical supplies are not guaranteed to any nation*. *There's a lot of politics and economics involved in healthcare decisions that are not necessarily directed at mutual utilitarian benefits of pharmaceuticals*, *in terms of patient outcomes*. *But having a list of essential medicines acknowledged can help direct our society to develop infrastructure and supply chains to protect the most important medicines*.*” (Participant 6*, *Healthcare Provider)*
	Stakeholders involved in providing medicines in the supply chain prioritise medicines according to different principles	*[Q12] “1) Medical relevance*. *Is this product a life-saving {medicine} versus {a medicine for} long-term disease improvement {or} symptomatic relief issue*? *The more life-saving {the medicine}*, *it becomes more essential*. *2) Demand*. *If {a medicine} has either a sporadic or responsive demand*, *it becomes an essential product we need to plan for*. *Flu vaccines are an essential medicine*. *It improves health outcomes*, *but because {of} it's sporadic demand you need to plan for its use*. *3) Supply*. *Who else can deliver this product*? *If there's four competitors of a product we don't see it as an essential medicine as a pharma company*. *It may be an essential medicine for a practising pharmacist but that's where the differentiation starts to occur*. *4) To deliver value to shareholders {as} a publicly listed company {and to} sell {medicine} at a positive margin*. *Some items within our portfolio we sell a lot of which makes a lot of money*, *and therefore it's a commercially essential medicine*.*” (Participant 8 –Pharmaceutical Industry)*
**3) Tensions amongst stakeholders are created by differing views on the function of an EML and conflicting interests surrounding the selection of medicines.**	The notion of essential medicines is highly confounded.	*[Q13] “What makes a drug essential*? *You can lay down some criteria*, *but they're not absolute and definitive*. *You can prioritise and heavily weigh them with a declining degree of weight*. *Then there's a transition between at what point {is} something essential or non-essential*. *That is highly confounded*. *{Clotrimazole for treating} candidiasis is an example*. *Not essential*. *Damn*! *Who says it's not essential*? *Someone has made that determination—in this case*, *other than the consumer*. *The government made the decision they were available over the counter*, *therefore we {society} wouldn't pay for them*. *Does that make them non-essential*? *Or they're available over the counter*? *What is the link between subsidy and essential medicines*?*” (Participant 11-Government)*
	The influence of cost containment is interpreted differently amongst stakeholders. Lowering costs of some medicines allow for expansion of the PBS to include more medicines	*[Q14] “One of the reasons the industry has supported all the price cut policies they renegotiated with government*.* *.* *. *is that they are designed to drive down the price of old drugs so that the health system could afford to list the new drugs coming through in the future*. *The Government {has} got the savings*, *now we can afford to bring {other} things on*.*” (Participant 5*, *Pharmaceutical Industry)*
	The PBS keeps medicine costs affordable for Australians despite high prices set by pharmaceutical companies	*[Q15] “The PBS has helped {so Australians} can afford all these new medicines*. *Drug companies might complain that the {price listed on the} PBS is too cheap*, *that they can't afford to sell medicines to {consumers at such a price}*. *It doesn't seem to stop them from registering their products and listing them on the PBS*.*” (Participant 1-Healthcare provider)*
	Demand for improved transparency around the pharmaceutical industry’s role and influence in the decision making process	*[Q16] “{The PBS has} got too many alternatives*.* *.* *. *You have to look at the make-up of the Pharmaceutical Benefits Advisory Committee (PBAC)*. *I believe the current PBAC membership has the necessary expertise and looks appropriately comprehensive*. *{But} drug companies can exert influence directly or through consumers who then pressure MPs (Members of Parliament)*. *I believe they could be under a lot of pressure to put new things on the list which probably should not be on the PBS*. *It is important that there are mechanisms to deal with that pressure*.*” (Participant 27*,*Academic)*

### 1. The definition and function of an EML in Australia was interpreted differently amongst stakeholders

All participants considered access to essential medicines as a basic human right that should be upheld by society [Q1]. However, most participants noted that in principle, the notion of an “essential” medicine in a HIC like Australia has evolved in terms of the definition and intended application of an EML [Q2]. Some participants considered the reimbursement of medicines through the Australian PBS was akin to a functioning EML [Q3]. However, many participants argued they could not distinguish between reimbursed medicines and essential medicines [Q4] [Q5]. While some believed an EML approach could support stakeholders in the supply chain to sustain fundamental health services [Q6], one participant highlighted that the consideration of a medicines’ cost and cost-effectiveness were contentious issues that undermined the notion of essential [Q7].

### 2. The selection criteria of medicines and the context to which the EML concept was applied were dynamic variables that influence decision making

Some participants reflected the selection of medicines for reimbursement has been increasingly driven by consumer needs [Q8]. Furthermore, some participants explained reimbursement decisions were driven by cost effectiveness and additional benefit of a medicine for some individuals rather than the essential need of a medicine by the population [Q9]. Many participants found it challenging to apply the concept of EML to the Australian context because EMLs were perceived to be useful for resource scarce settings [Q10]. However, some participants regarded essential medicines as part of a complex pharmaceutical supply chain. Even in HICs, access cannot always be guaranteed [Q11]. One participant described the characteristics of medicines that are categorised as “essential” by the pharmaceutical industry within the supply chain [Q12].

### 3. Tensions amongst stakeholders were created by differing views on the role of an EML and conflicting interests surrounding the selection of medicines

The selection and supply of reimbursed medicines were complex issues. One participant reflected that the notion of essential medicines was highly confounded [Q13]. The influence of cost containment was interpreted differently amongst stakeholders. Some participants thought decisions about whether a medicine was listed on the PBS included consideration of cost containment by lowering prices of generic medicines in order to expand the PBS to list new medicines [Q14]. While some participants felt that despite high prices set by pharmaceutical companies, the PBS has helped keep medicine costs affordable for Australians [Q15]. Many participants encouraged more transparency around the pharmaceutical industry’s role and influence in the decision making process [Q16].

## Discussion

This study found that stakeholders had a broad range of views surrounding the application and/or relevance of the EML concept within the Australian context. The findings illustrated a diversity of perspectives amongst stakeholders, often reflecting their position within the health care system. Views held by consumers and health professionals with respect to what was “essential” and what medicines should therefore be accessible and reimbursed, were sometimes in contrast to the views of supply chain managers or policy makers. This demonstrated the tussle between perceptions of an EML based on a utilitarian approach in the health system for a population versus an EML established to meet individuals’ needs.

Stakeholders’opinions of what constituted an ‘essential medicine’ differed depending on whether they were being tasked with making decisions for the population or they were considering a medicine to be ‘essential’ for an individual. From a consumer’s perspective, a medicine is considered essential at the point of care. Education can empower consumers to request access to medicines they deem essential. Individuals want access to the right drug, at an affordable price, of safe quality and available at all times within close proximity [40]. On the other hand, policy makers within the health system consider medicines to be essential based on a country’s priority health care needs and cost effectiveness, in order to facilitate wider access to medicines by the population [12]. Although the notion of ‘essential’ for an individual or health system is often used interchangeably, results showed that the two perspectives are often divergent and therefore applied as different concepts.

While participants in this study supported the EML concept and were able to explain its general function, most found it difficult to determine what constituted an essential medicine due to the confounding nature of individual or utilitarian needs, as described above. Historically, an EML was once a basic formulary of survival and emergency medicines that adhered to utilitarian principles [41]. Today, the EML concept has evolved, influenced by human rights’ movements, disease specific epidemics and societal values, to become a much more complex list used to save and also improve the quality of life for many more individuals [1,41]. Furthermore, the evolution in pharmacotherapy from prescribing for a disease to prescribing for an individual includes consideration of those with multiple conditions. This could mean that standard treatment guidelines, which are usually limited to the treatment of a single condition and informed by data gathered in clinical trials, may not be applicable for patients with multiple co-morbidities. Adding to this complexity is the continual development of pharmaceutical products in the market, leading to more choices and alternatives available. This expansion and evolution of the “EML concept” has led to the development of extensive lists of publicly subsidised medicines in HICs, such as Australia. These lists often have multiple pharmacotherapy choices available to manage conditions [42,43]. While in some health systems, lists can be sub-categorised using “vital”, “essential”, or “non-essential” medicine (VEN) models of procurement to identify priority medicines [44]. In line with this trend of list expansion, the 19^th^ WHO EML includes a range of high cost medicines that address priority diseases in a variety of settings [4]. For example, Trastuzumab in breast cancer and Sofosbuvir for Hepatitis C [4,5]. Therefore, in HICs like Australia, it remains unclear if the decisions to add and manage medicines on these lists still adhere to the foundational utilitarian principles that were once crucial to its inception.

Study participants described the PBS accommodated broader individual needs than EMLs. Some highlighted that consumer engagement in the decision-making process has contributed to the wide breadth of the PBS to include sometimes rare and costly medicines to meet individual needs and expand access. The tussle between policy makers and consumer driven decisions may be explained by the Comprehensive Theory of Collaboration which describes three types of self-interest: shared, differing, and opposing [39]. **Shared self-interests** provide clear conditions for a health system to identify and manage national priority medicines. For example, the utilitarian-type public health program around influenza pandemic planning manages and ensures continued supply of vaccines and medicines to all Australians. Meanwhile, participants’ discussed how **differing interests** contributed to the wide breadth of reimbursement under the PBS, which can include multiple options within the same drug class. Lastly, tensions between stakeholders may result from individuals having **opposing interests**. Participants described these tensions in the deliberations involved in the selection of high cost medicines for reimbursement, and negotiations to lower prices of generic medicines. For example, cost containment may prevent few individuals from accessing rare high cost medicines through public subsidised funding. At the same time, pharmaceutical companies may have opposing interests to governments and consumers regarding pricing of their medicines, and may be pressured to lower the cost of products in order to have them listed and utilised. This demonstrates how individuals’ opposing and differing interests contribute to the expansion and costs of reimbursement lists in HICs, in contrast to shared (utilitarian) interests around priority medicines.

It is often assumed that an EML ensures access to prioritised medicines. Therefore, stakeholders perceived that applying the EML concept provided access to affordable medicines to individuals and the health system. Yet, even in a HIC like Australia, access to medicines can be hindered. Two examples of vulnerabilities highlighted by stakeholders include unforseen drug shortages and high costs of medicines. Despite reimbursement, essential medicines are part of a complex global supply network, in which supply cannot always be guaranteed during unpredictable drug shortages [45–47]. This was demonstrated during worldwide shortages of injectable benzyl penicillin in 2011 and morphine in 2013, causing much strain on the health system and consumers [48,49].

Secondly, participants described the affordability of medicines for consumers and health systems was a growing concern as pharmaceutical expenditures continue to rise [17]. Health systems may need to pay high prices for these medicines and governments must make difficult decisions as to how these medicines will be paid for. As a national reimbursement scheme, the PBS negotiates lower prices with manufacturers to provide, “timely access to medicines that Australians need, at a cost individuals and the community can afford” [26]. There are a range of therapeutic options available through the PBS, including rare and high cost medicines. Additional arrangements provide funding and restricted supply for medicines under: the Life Saving Drugs program, Section 100 (S100) program, or Special Access Scheme for rare or specialised conditions [50–53]. Despite this, Australia still pays some of the highest prices for generic medicines compared to other countries, and up to twenty times more than its neighbouring country New Zealand, where sole-sourcing and pooled procurement strategies have been applied to obtain lower prices [17,54,55].

Furthermore, consumers’ inability to afford out-of-pocket expenses can hinder access to medicines, sometimes leading to “catastrophic drug costs” [56,57]. Although Australia is a HIC, there is disparity of wealth across the population [58]. Therefore, whilst catastrophic drug costs are rare due to PBS subsidisation, out-of-pocket expenses (ie. co-payment of $37.70 AUD for general patients in 2015 [59], which increases annually), can become too much for some individuals to afford, especially for those on regular multiple medicines [17,60]. Meanwhile, some medicines deemed essential to individuals and listed on the WHO EML, but may not be reimbursed under the PBS, leading to out-of-pocket payments by consumers. It should be noted that in Australia, PBS listed medicines are indirectly paid for by all individuals through the government income-based taxation system. Additionally, individuals requiring medicines can pay for them through direct out-of-pocket payments (usually for medicines not listed on the PBS or those that are priced under the co-payment amount), or through co-payments for government subsidised medicines. Some may also receive partial reimbursement for high cost, non-listed medicines through private health insurance. Similar to Cameron et al. [9], HICs also face challenges to guarantee supply of generic medicines and high prices. Therefore solutions and approaches in HICs may provide useful to LMIC settings.

All participants acknowledged the PBS as a national reimbursement scheme generally effective at meeting Australians’ health needs. However, the issues raised above, led them to explore strategies from the EML concept to prioritise medicines in the supply chain. In line with Hogerzeil [6] and Wood & Gray [39], applying collective (utilitarian) interests to identify a core list of medicines needed to maintain the basic functions of a health system, can improve resilience to supply disruptions and manage rising medicine expenditure. Furthermore, in a US study, Millar et al. [61] found that WHO EML medicines appeared on most Preferred Drug Lists (PDLs), suggesting that the EML could function as a core list for PDL development to guide procurement and decrease prices of medicines.

The comprehensive theory of collaboration was applied as the underpinning theory to explain the variability in the notion of what is meant by an essential medicine. “Self-interests” in collaboration surrounding EMLs influence stakeholders’ views of what they deemed essential. Hence, multiple factors may influence these self interests which guide individuals to choose medicines, as represented in S1 Fig. This conceptual model supports that an EML is used at a health system level, for most people, most of the time. However, for individuals at the point of care with many options, an EML is often viewed as not relevant. Although, a specific drug on the EML may be appropriate. The layers in S1 Fig demonstrated the influence of self interest at different levels of care. As one moves through the levels of the health system from government policies towards the point of care, the notion of self-interest becomes more focused on the individual and less utilitarian. This phenomenon has contributed to the expansion of reimbursement lists. In contrast, when a medicine becomes unavailable, where there are no alternatives or options are unaffordable for individuals or governments, then this focus shifts back towards shared priorities in order to ensure the delivery of health services. Hence, this conceptual model demonstrates the concept of EML becomes fragmented as it moves through the pharmaceutical supply chain towards the point of care.

A health system is unable to meet every need of all individuals within a population. Thus, S1 **Fig** illustrates the disparity between health systems that focus reimbursement schemes on utilitarian population needs versus meeting the expanding needs of individuals. This is most apparent between LMIC and HIC approaches to reimbursing medicines. When consumers absorb the difference in out-of-pocket medicine costs outside of government reimbursement programs, it can sometimes become exorbitantly unaffordable [57]. Therefore, consumers’ opposing interests remaining outside population wide reimbursement schemes may need alternative funding assistance or education programs to address ongoing tensions.

### Strengths and Limitations

The results in this study were not meant to be generalisable. Instead, the use of qualitative methodology allowed researchers the opportunity to explore issues facing decision makers when creating national medicines lists. The strength of this study lies in offering an in-depth exploration of a broad range of key stakeholder views through rich qualitative data. This study gathered the perspectives of leaders and senior management throughout the continuum of a complex supply chain involved in decision-making surrounding medicines management in HICs. The study however, did not explore perspectives of primary health care workers, which should be pursued in future studies. It showed there were a variety of views as to what the term EML really means and how it relates to policy in a HIC context. While not included in the scope of this study, future studies could examine how this can be applied in LMICs. Additionally, future studies could examine further each individuals work environment and its effect on their views.

## Conclusions

The EML concept is simple, idealistic, and has been widely received. However, this study showed that the notion of “essential” is not implicit. Although beneficial in theory, Australian stakeholders struggled to identify how the EML concept functioned in practice. In Australia, decision making around reimbursement of medicines has strayed from the fundamental utilitarian concept of essential medicines. Instead, focus is on cost-effectiveness of new technologies and meeting unmet individual needs through expansive reimbursement lists. Interestingly, many of those involved in medicine reimbursement decisions and management of the pharmaceutical supply chain did not consider the EML concept in their approach. Moreover, the results of this study challenge for whom we consider essential medicines for, and if the term essential is currently appropriate. Therefore, this challenged whether the EML concept was out-dated or underutilised in HICs such as Australia. As medicine expenditures continue to rise worldwide and global drug shortages remain frequent and problematic, the EML concept can potentially play a role in managing health resources. Therefore, further investigation is required to address innovative ways to apply EML concepts in HICs to support population wide access to prioritised medicines, while strengthening collaborations between pharmaceutical supply chain stakeholders. Transitioning the EML concept from policy to practice continues to be a work in progress.

## Supporting Information

S1 Fig Conceptual Model. A core list of medicines reflects shared stakeholder interests to support the fundamental basic needs of a country’s health system. Bearing in mind that all medicines on the WHO EML are not assumed to be included on a country’s EML, since they are adapted to meet each health system’s needs. In contrast, broader reimbursement schemes have wider inclusion of differing and opposing interests between stakeholders. The extent of out-of-pocket expenses individuals may incur beyond the shared priorities supported by the health system are illustrated by the difference in area from the outer layer. This model does not take into consideration that a health system with broader reimbursement may also be at risk of reimbursing inappropriate medicines. Also, countries without a national EML (ie. the US) are not illustrated in this model despite high out-of-pocket expenses, due to the high variability of private insurance schemes. And while some health systems do not have EMLs, people can still access medicines if they are willing and able to pay-out-of pocket or have alternative funding assistance available such as private insurance.
(TIF)

S1 Text Qualitative Semi-structured Interview Protocol. (PDF)

S2 Text COREQ-32 item checklist for reporting qualitative studies. (PDF)
