# Supplementary figures and images for: Essential Medicines in a High Income Country: Essential to Whom?
Source: PLoS One. 2015 Dec 9;10(12):e0143654. doi: 10.1371/journal.pone.0143654 (PMC4674059; doi:10.1371/journal.pone.0143654)

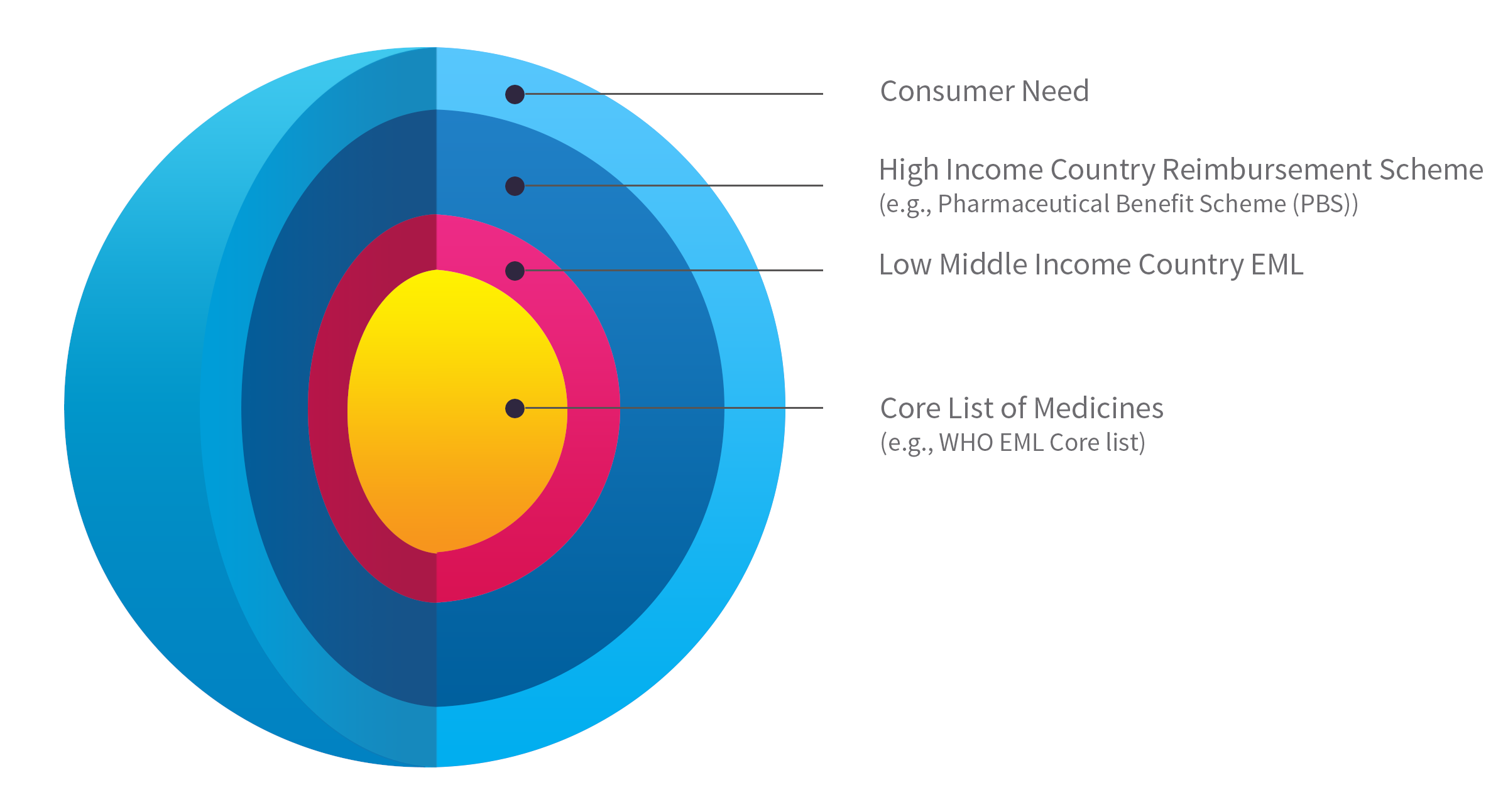

Supplement: S1 Fig — A core list of medicines reflects shared stakeholder interests to support the fundamental basic needs of a country’s health system. Bearing in mind that all medicines on the WHO EML are not assumed to be included on a country’s EML, since they are adapted to meet each health system’s needs. In contrast, broader reimbursement schemes have wider inclusion of differing and opposing interests between stakeholders. The extent of out-of-pocket expenses individuals may incur beyond the shared priorities supported by the health system are illustrated by the difference in area from the outer layer. This model does not take into consideration that a health system with broader reimbursement may also be at risk of reimbursing inappropriate medicines. Also, countries without a national EML (ie. the US) are not illustrated in this model despite high out-of-pocket expenses, due to the high variability of private insurance schemes. And while some health systems do not have EMLs, people can still access medicines if they are willing and able to pay-out-of pocket or have alternative funding assistance available such as private insurance. (TIF) [file pone.0143654.s001.tif]
