# Supplementary material for: Essential Medicines in a High Income Country: Essential to Whom?
Source: PLoS One. 2015 Dec 9;10(12):e0143654. doi: 10.1371/journal.pone.0143654 (PMC4674059; doi:10.1371/journal.pone.0143654)
Supplement: S1 Text — (PDF) [file pone.0143654.s002.pdf]

THE UNIVERSITY OF SYDNEY

Interview Guide

Title: The Management and Supply of Essential Medicines

Country: ____
Occupational Setting: ________________________________
Profession: ____
Number of Years Practiced: ____________________________
Gender: M / F
Age: _____________
PBI Code: ___________
Date: ________________________
Time Started:_________ **Time Completed:** _____________
Location: ____
Interaction Type: **Face-to-Face** **Teleconference** **Skype**

The Role and Application of the Essential Medicines List:

1. Are you familiar with the Essential Medicines List in your country?
2. What does the concept of having an Essential Medicines List mean to you?
3. What makes a drug essential?
4. How is the concept of the Essential Medicines List applied in your practice setting?
5. Please describe examples of effective use of the Essential Medicines List in your practice setting.
6. Please discuss some barriers to the effective use of the Essential Medicines List in your practice setting.

The Appropriate use of the Essential Medicines List:

7. What factors influence how essential and non-essential medicines are used in your practice setting?
8. How does the essential medicines list affect your practice?

The Availability of the Essential Medicines List:

9. Have you encountered any difficulties obtaining any medicines from the Essential Medicines List? Please describe your experience.
10. Please describe the availability of medicines from the Essential Medicines List in your practice setting.

The Affordability of the Essential Medicines List:

11. How does the Essential Medicines list effect costs for individuals, health care professionals, health facilities/institutions, governments, and pharmaceutical manufacturers?

The Quality of Medicines from the Essential Medicines List:

12. Please describe the quality of essential medicines available in your country.

Other:

13. How does the essential medicine list effect patients and their health care experience?
14. In your opinion, what are the key issues surrounding the Essential Medicines List?

Additional Comments:
