# Supplementary material for: Essential Medicines in a High Income Country: Essential to Whom?
Source: PLoS One. 2015 Dec 9;10(12):e0143654. doi: 10.1371/journal.pone.0143654 (PMC4674059; doi:10.1371/journal.pone.0143654)
Supplement: S2 Text — (PDF) [file pone.0143654.s003.pdf]

Consolidated criteria for reporting qualitative studies (COREQ): 32-item checklist

Developed from:
Tong A, Sainsbury P, Craig J. Consolidated criteria for reporting qualitative research (COREQ): a 32-item checklist for interviews and focus groups. *International Journal for Quality in Health Care*. 2007. Volume 19, Number 6: pp. 349 – 357

No. Item	Guide questions/description	Reported on Page #
**Domain 1: Research team and reflexivity**		
Personal Characteristics		
1. Interviewer/facilitator	Which author/s conducted the interview or focus group?	Mai Duong
2. Credentials	What were the researcher's credentials? E.g. PhD, MD	Mai Duong, BSc.Pharm,BHSc(Hons), RPh, M.Phil Candidate<br><br>Rebekah J. Moles, PhD,DipHPharm,BPharm, Senior Lecturer<br><br>Betty Chaar, PhD,MHL,BPharm, Senior Lecturer<br><br>Timothy F. Chen, PhD,DipHPharm,Pharm, MPS,MSHP,Associate Professor
3. Occupation	What was their occupation at the time of the study?	Pharmacist, M.Phil student, Teaching Assistant
4. Gender	Was the researcher male or female?	Female
5. Experience and training	What experience or training did the researcher have?	Methods -<br>The researcher gathered data from hospital interviews in Costa Rica in a study at the University of Toronto in 2006. She participated in ACSPRI qualitative research methods training courses in Australia in 2013.
Relationship with participants		

6. Relationship established	Was a relationship established prior to study commencement?	No
7. Participant knowledge of the interviewer	What did the participants know about the researcher? e.g. personal goals, reasons for doing the research	Participant information sheet and Consent Form
8. Interviewer characteristics	What characteristics were reported about the interviewer/facilitator? e.g. Bias, assumptions, reasons and interests in the research topic	Methods
**Domain 2: study design**		
Theoretical framework		
9. Methodological orientation and Theory	What methodological orientation was stated to underpin the study? e.g. grounded theory, discourse analysis, ethnography, phenomenology, content analysis	Methods
Participant selection		
10. Sampling	How were participants selected? e.g. purposive, convenience, consecutive, snowball	Methods
11. Method of approach	How were participants approached? e.g. face-to-face, telephone, mail, email	Methods
12. Sample size	How many participants were in the study?	Methods
13. Non-participation	How many people refused to participate or dropped out? Reasons?	Methods
Setting		
14. Setting of data collection	Where was the data collected? e.g. home, clinic, workplace	Methods
15. Presence of non-participants	Was anyone else present besides the participants and researchers?	No
16. Description of sample	What are the important characteristics of the sample? e.g. demographic data, date	Methods
Data collection		
17. Interview guide	Were questions, prompts, guides provided by the authors? Was it pilot tested?	Methods
18. Repeat interviews	Were repeat inter views carried out? If yes, how many?	No
19. Audio/visual recording	Did the research use audio or visual recording to collect the data?	Methods
20. Field notes	Were field notes made during and/or after the inter view or focus group?	Methods
21. Duration	What was the duration of the inter views or focus group?	Methods
22. Data saturation	Was data saturation discussed?	Methods
23. Transcripts returned	Were transcripts returned to participants for comment and/or	Methods

	correction?	
**Domain 3: analysis and findings**		
Data analysis		
24. Number of data coders	How many data coders coded the data?	Methods
25. Description of the coding tree	Did authors provide a description of the coding tree?	Methods
26. Derivation of themes	Were themes identified in advance or derived from the data?	Methods
27. Software	What software, if applicable, was used to manage the data?	Methods
28. Participant checking	Did participants provide feedback on the findings?	Methods
Reporting		
29. Quotations presented	Were participant quotations presented to illustrate the themes/findings? Was each quotation identified? e.g. participant number	Results
30. Data and findings consistent	Was there consistency between the data presented and the findings?	Discussion
31. Clarity of major themes	Were major themes clearly presented in the findings?	Results
32. Clarity of minor themes	Is there a description of diverse cases or discussion of minor themes?	Results
